# Genetic evaluation of a selective breeding program for common carp *Cyprinus carpio* conducted from 2004 to 2014

**DOI:** 10.1186/s12863-015-0256-2

**Published:** 2015-07-29

**Authors:** Zaijie Dong, Nguyen Hong Nguyen, Wenbin Zhu

**Affiliations:** Freshwater Fisheries Research Centre of Chinese Academy of Fishery Sciences, Key Laboratory of Freshwater Fisheries and Germplasm Resources Utilization, Ministry of Agriculture, Wuxi, 214081 China; University of the Sunshine Coast, Locked Bag 4, Maroochydore, DC QLD 4558 Australia

**Keywords:** Genetic improvement, Selection response, Heritability and common carp

## Abstract

**Background:**

The study evaluated genetic progress of a breeding program for common carp undergoing four generations of selection for increased harvest body weight from 2004 to 2014. The pedigree included 17,351 individual fish which were offspring of 342 sires and 352 dams. Genetic parameters for body weight at about two years of age and survival rate during grow-out period were also estimated using the residual maximum likelihood method applied to a two-trait linear mixed model. Direct response in body weight and correlated changes in survival were measured as the differences in: i) estimated breeding values (EBVs) between the two lines; and ii) EBVs of the selection line between successive generations.

**Results:**

Direct gain in body weight ranged from 0.20 to 0.90 genetic standard deviation units and averaged 7 % of the base population per generation (two years per generation). Correlated changes in survival were negligible, indicating that the selection program for high growth did not have any adverse effect on this trait in the present population. The heritability for body weight was moderate (0.17, s.e. 0.05), whereas the estimate for survival was low (0.05–0.17) but significantly different from zero across linear mixed and threshold generalised statistical models. Our results predict that body weight or/and other growth related traits will continue to respond to selection and that there is potential to improve survival through direct genetic means. Correlated improvement in survival to selection for increased body weight was hardly achieved, as the genetic correlation between the two traits was not different from zero.

**Conclusions:**

It is concluded that selection for increased harvest body weight resulted in significant improvement in growth performance of the present population of common carp *Cyprinus carpio*.

**Electronic supplementary material:**

The online version of this article (doi:10.1186/s12863-015-0256-2) contains supplementary material, which is available to authorized users.

## Background

Common carp (*Cyprinus carpio* L.) is one of the most economically important freshwater species for aquaculture in the world including China [1]. In China it is the third important cultured finfish, alongside grass carp (*Ctenopharyngodon idellus*), silver carp (*Hypophthalmichthys molitrix*) and bighead carp (*Hypophthalmichthys nobilis*). In 2011, the production of cultured common carp reached 2.71 million tonnes and accounted for 11.0 % of the total inland aquaculture production in China [2].

*C. carpio* is widely distributed throughout China and shows many morphological and genetic variations after local artificial breeding and natural selection. The most important wild and domesticated common carp populations in China include Huanghe carp (*C. carpio haematopterus* Temminck et Schlegel), Heilongjiang carp (*C. carpio haematopterus*), purse red carp (*C. carpio var. wuyuanensis*) and Xingguo red carp (*C. carpio var. xingguonensis*). China has a long history of culturing common carp and the species has played a significant socio-economic role in the society [3]. However, breeding this species in captivity only began in 1970’s when some varieties were developed for aquaculture, mainly by crossbreeding technique [4]. Jian carp (*C. carpio* var. *jian*), which was bred by scientists in the Freshwater Fisheries Research Center (FFRC) of Chinese Academy of Fishery Sciences in 1990s [4], is the first variety of common carp that was produced through artificial breeding. The techniques used to produce this variety include hybridization, within-family selection and gynogenesis. The gross production of Jian carp is about 30 % higher than other varieties of common carp [4].

Due to the superiority in production characteristics of the Jian carp, a family selective breeding program to further improve quality and growth performance of this strain has been conducted at FFRC since 2004. We applied the method used for other aquatic animal species by establishing a synthetic base population from different sources and using mixed model methodology to rank selection candidates based on their estimated breeding values. This enabled us to select superior animals to become parents of the next generation [5]. This approach has been successfully applied to improve productivity of several terrestrial farmed animals [6] and aquaculture species [7]. Experimental studies with common carp report that selection for increased body weight also resulted in significant improvement in growth performance [8] and was correlated with increases in body traits (e.g. body length, depth or width) [9]. However, possible changes in fitness related traits, such as survival during grow-out, have not been reported.

The aim of this paper was to conduct genetic evaluation of the breeding program for common carp after four generations of selection in order to estimate genetic parameters and selection response for body weight and survival. Linear mixed and generalised threshold logistic models were used to estimate heritability for body weight and survival, respectively. The response (or genetic gain) was measured as the difference in estimated breeding values (EBVs) between the selection line and control group.

## Methods

### Experimental location

The breeding program for common carp was conducted at the Freshwater Fisheries Research Centre (FFRC) of the Chinese Academy of Fishery Sciences (CAFS), following The Guide for the Care and Use of Experimental Animals of China.

FFRC is located in Wuxi city besides the third largest freshwater lake (Lake Tai) and is about 30 km to the south of the Yangtze River. The annual temperature ranges between −7 and 38 °C (average 15.5 °C) and the average annual rainfall is approximately 1000 mm. Topographically, FFRC is located in low plain areas (200 m above sea level) along the lower reaches of the Yangtze River. The freshwater pond, pH 7.2–8.5, has a salinity level below 0.2 ppt.

### Origin of the common carp population

The synthetic base population was formed from a complete diallel cross involving the Jian carp and Huanghe carp strains produced in April 2004. A total of 78 families were produced, tagged and reared for 20 months. Measurements of body weight, length and height were made on all individual fish. Data from this base population were analysed to estimate breeding values. These were used to carry out during the first round of selection. In 2006, a mating protocol to form the selection and control lines was designed based on the analysis of growth data of the 78 families produced in 2004. A total of 81 pairs of fish spawned successfully for the selection line, whereas 19 pairs of fish spawned for the control line. Their progeny were contemporaneously reared by family under the same pond conditions. In 2008, the third strain of Heilongjiang carp (20 females, 1.85 kg and 20 males, 1.5 kg) was incorporated into the population and 66 selection families were established.

### Family production and rearing

#### Mating design and family production

Parental breeders in each generation were paired on the basis of their estimated breeding values (EBV) and their genetic relationship with other individuals in the pedigree. Sexual maturity of the breeders was examined externally as common carp normally reach maturation in the spawning season (April-May). Induced spawning was applied to produce full- and half-sib families. A hormone injection was given to parental fish: females were injected with 500 IU HCG + 4 μg LRH-A2 per kg; males 250 IU HCG + 2 μg LRH-A2 per kg. Spawning began around 12 h later.

Each pair of the injected fish was released into an individual hapa (1 × 1 × 1 m^3^, 16 mesh per cm) installed in the two earthen ponds. The fish spawned naturally (without outside intervention) in the hapas.

Twenty four hours after the hormone injections, the cages and eggs were transferred to the egg-hatching and fry-rearing site in an earthen pond (0.35 ha, 2 m deep). The hatching hapas were of 1 × 1 × 1 m^3^ (20 mesh per cm) installed in indoor cement tanks (70 m^3^). Twenty hapas were installed in each tank. Eggs hatched after 48 ± 2 h. Eggs from each family were separately hatched in different hapas.

#### Family rearing procedures

Each full sib group was reared in the same hapa (1 × 1 × 1 m^3^) for 7d before being transferred to a cage with larger mesh size (8 mesh per cm). The stocking density in the cage was 1000 larvae per m^3^. Fry were not counted immediately after hatching due to high fecundity of the species. Soybean milk and fine granular formulated diet were used as the feed for the fry and provided twice a day.

When fish larvae reached the length of approximately 3 mm (after about 2 months after hatching), they were transferred to cages 1 × 1 × 1 m^3^ with larger mesh size (3 mm). The stocking density was reduced to 100 pieces per m^3^. For each family, only 100 randomly sampled individuals were retained and restocked in the new cage. The fish were reared in the same cage for about two months and were fed twice a day with pellet feed until tagging. The feeding rate was from 2 to 4 % of the body weight, adjusted according to ambient temperature. The diet had 32 % protein and 3 % lipid.

Offspring of each family were reared in separate cages until tagging was completed. All the cages were installed in one pond to ensure uniform rearing conditions. The cages were cleaned and checked for netting once a week during the rearing period.

#### Individual tagging

The fish were tagged after 2 months of rearing at an average body weight of about 20 g. The tagging of all the families was completed within two days. In each family, a random sample of 50 fingerlings were tagged using Passive Integrated Transponder (PIT) for individual identification.

All the tagged fish were kept in indoor cement tanks (72 m^2^) for 1–2 weeks to monitor mortality before releasing them into an earthen pond for further rearing. The stocking density was 20 fingerlings per m^2^. Normal feeding was resumed one day after tagging. During this period a small number (~1 %) of fish died or lost their tag (about 0.1 %) and these were replaced by their siblings from the same family.

### Communal testing

Communal testing of all families was conducted at two different stages over a period of 11.5–13.5 months. In the first period/year, all the tagged fish were released in one earthen pond of 0.16 ha and a depth of 1.8 m, after the temporary holding. The fish were daily fed with pellet feed (28 % protein and 3 % lipid). Feeding was practiced twice a day, during early morning and late afternoon. The stocking density in this period was about 18,000 fish per ha. The water temperature was below 25 °C.

In the second period, the fish were reared in the new pond (0.34 ha) with a water depth of 2.0 m for 6 months until the second sampling. Feed (28 % protein) was provided twice a day, once in the early morning and once in the late afternoon throughout the culture period. The feeding rate was between 1 and 4 % of the estimated body mass. Some mortality occurred in the hot season. The dead fish were collected and their tags were recovered.

The stocking density in this period was 15,000 tagged fish per ha, together with 2000 silver carp per ha and 1000 bighead carp per ha. Stocking of silver carp and bighead carp aimed to regulate the water fertility. The water temperature during this period (January to December) ranged from 4 to 32 °C.

### Harvesting

Harvesting and measurement were conducted every twelve months of culture and after about one and a half years from birth (482 d in G2 and G3 and 557d in G4). The fish were harvested through total drainage of the pond because common carp are difficult to catch by netting. All the harvested fish were scanned and measured, including body weight, body length and body height as well as their tag identification and sex. Only a small proportion of the tagged fish (0.009 % or 27 fish) were not identified because: (i) fish had lost their tags during the rearing; (ii) tags were defective; or (iii) fish died during the rearing period and were not collected before sinking to the bottom and decomposing.

### Selection procedures

Linear animal mixed model analyses were performed each generation to estimate the breeding value (EBV) for body weight of individual fish. Based on individual and family rankings by EBV, best (highest EBV) fish were selected to become parents in the selection line, whereas the control group was selected based on the EBV mean of the population. Two to three times more fish than the actual number of breeders were selected as candidates for the selection line and control group. The mating protocol was designed with a restriction on the number of fish per family contributed to the next generation. The mating of close relatives was also avoided. These fish were kept in an earthen pond and provided a high quality diet.

In each generation, the mating pairs consisted of 90 for the selection and 20 for the control. The selected male and female breeders were kept in separate earthen ponds to avoid any possible natural breeding. Feeding, management and induced breeding were practised as details above. The majority of the mating pairs spawned 48 h after the injection. A small number of pairs (3–10 %) failed to spawn across the generations. Some of them had high EBV ranking. They were held in indoor tanks (males separated from females) and fed a high quality brooder diet. Following the second hormone injection, about 60 % of the pairs that initially failed spawned. High mortality also occurred at hatching in 2–4 families each generation (less than 100 fry survived out of five to 10 thousands), probably due to poor egg quality.

In total, 66–84 selection and 16–19 control families were successfully produced across generations. Fry nursing/rearing, tagging, communal grow-out and harvest data were recorded as described in the above sections. Genetic evaluation was then conducted and a new breeding cycle was repeated every two years (i.e. generation). The same selection procedures, animal husbandry and management regimes were practised in all generations. A summary of the production cycle from mating to harvesting in common carp is given in Additional file 1.

**Table 1:** The number of sires, dams and offspring for the selection line and control group in four generations of selection

Generation	Year	Line	Sire	Dam	Offspring
1	2004 – 2005	Base population	78	78	2911
2	2006 – 2007	Selection	84	84	4266
		Control	19	19	949
3	2008 - 2009	Selection	66	73	3886
		Control	14	13	817
4	2012 - 2013	Selection	72	72	3683
		Control	16	16	813
Total	All years		342	352	17,351

### Statistical analysis

Genetic parameters for body weight were estimated using linear mixed model [Eq 1] that included the fixed effects of generation (*G*_*i*_ = 4), line (*L*_*j*_ = 2, selection and control), sex (*S*_*k*_ =2, female and male) and their two-way interactions between these factors. Stocking weight (W_*l*_) within line and generation was also fitted as a linear covariate in the model. The random terms were the additive genetics of individual fish (*a*_*m*_) and common full-sibs (*c*_*n*_).

1 $$ {y}_{ijklmn}=\mu + {G}_i + {L}_j + {S}_k + G\times {L}_{ij} + G\times {S}_{ik} + L\times {S}_{jk} + {W}_l\left(G,\ L\right) + {a}_m + {c}_n + {e}_{ijklmn} $$

where *y*_*ijklmn*_ is the trait observation, *G*_*i*_*, L*_*j*_*, S*_*k*_*,, W*_*l*_*, a*_*m*_ and *c*_*n*_ are as defined above and *e*_*ijklmn*_ is the error term

As survival was recorded as a binary expression (fish that survived at harvest were coded as 1, *n* =11966 and those absent at harvest were coded as 0, *n* =3765), this trait was analysed using the threshold generalised logistic sire model [10]. Under the logistic model, calculation of heritability for survival assumed that residual variance was corrected by π^2^/3 (3.289) factor. In addition, a standard animal mixed model was used to estimate heritability for this trait. Both models used to analyse survival rate included the fixed effects as described in Additional file 2.

Phenotypic and genetic correlations were obtained from a bivariate animal mixed model. All the analyses were conducted using ASReml version 3.0 [11].

Selection responses for both body weight and survival were measured as the difference in estimated breeding values (EBVs) between the selection line and control group or between successive generations. The direct genetic gain for body weight and correlated changes in survival were expressed in actual units (gram for body weight and % for survival), genetic standard deviation unit (SD_A_) and percentage of the base population. The statistical model used to estimate EBVs for body weight and survival were the same as those used to estimate the heritability.

## Results

### Characteristics of the population and data

Over four generations of selection from 2004 to 2014, a total of 17,351 offspring produced from 342 sires and 352 dams were performance tested in earthen pond over/during an average grow-out period of 383 days. The number of offspring and their parents (sires and dams) in each generation is given in Table 1.

At final harvest (383 days), the number of fish with the data for body weight and survival are shown in Table 2 together with basic statistic parameters for these traits. The average body weight of the population at final harvest was 0.9 kg and survival rate during grow-out was 74 %. This population showed a large variation in body weight as shown by the high coefficient of variation of 70 %.

**Table 2:** Basic statistics for traits studied

Traits	Unit	N	Mean	SD	CV (%)
Weight	g	11680	944.7	657.4	69.6
Survival	%	17,351	73.9	43.9	59.4

The analysis of variance using the general linear model showed that the main effects of generation, line and sex were statistically significant for body weight. The two-way interactions among these factors (except for line × sex), as well as the linear covariate of stocking weight within line and generation, were also significant for this trait, i.e. body weight (P < 0.001). However, none of these effects was significant for survival when the generalised linear model was used (Additional file 2).

### Least squares means for body weight and survival of the selection line and control

Least squares means (LSMs) for body weight and survival were obtained using the linear mixed model and the threshold generalised logistic model, respectively. They are presented for each generation in Table 3. The selection line had significantly greater body weight than that of the control in all generations from 2004 to 2014 (P < 0.05 to 0.01). However, the difference in LSMs for survival between the selection line and control group was not significant (P > 0.05).

### Sexual size dimorphism

**Fig. 1:**
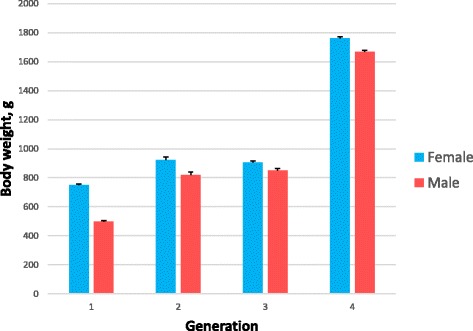
Least squares means of body weight by sex in four generations of selection

Sexual size dimorphism (SSD) occurred in the early phase of growth. The difference in body weight of fingerlings at stocking was small (2 %) but significant (P < 0.05). After 1 year of culture from hatching, female common carp had 34.4 % greater body weight than that of male (P < 0.01). At final harvest (one and a half to 2 years from birth) the between-sex difference in body weight was statistically significant (P < 0.001) in all generations (Fig. 1). In contrast to body weight, survival during grow-out did not differ between female and male common carp during the course of the selection program (Additional file 2).

### Heritability, common environmental effects and correlations

Estimates of heritability for body weight and survival and the phenotypic and genetic correlations between the two traits are given in Table 4. Body weight is moderately heritable (h^2^ = 0.17 ± 0.05) and the common full-sib effect (c^2^) accounted for 16 % of the total phenotypic variation (c^2^ = 0.16). The estimates of heritability for survival, estimated from linear animal mixed model and threshold logistic sire, were low (0.05 ± 0.01) and moderate (0.17 ± 0.03), respectively. However, both estimates were significant, based on their low standard errors.

**Table 3:** Least squares means (± se) for body weight (g) and survival (%) in the selection line and control group

Trait	Generation	Control	Selection	% difference
Weight	G2	575.7 ± 9.1	590.8 ± 4.3	2.62
	G3	701.8 ± 9.5	747.2 ± 4.5	6.47
	G4	2085.1 ± 11.2	2154.2 ± 5.3	3.31
Survival	G2	71.0 ± 1.47	69.1 ± 0.71	−2.89
	G3	74.4 ± 1.53	71.3 ± 0.73	−4.22
	G4	58.7 ± 1.73	60.0 ± 0.81	2.32

### Selection response

Genetic response to selection was measured as the difference either in estimated breeding values (EBVs) between the selection line and control (Table 5) or those between successive generations (Table 6). For each method, the results were presented in actual unit of measurements (g for body weight and % for survival), genetic standard deviation unit (SD_A_) and percentage of the base population. Regardless of expression units, the estimates of direct genetic gain for body weight and correlated response for survival were consistent between the two methods. For body weight, selection achieved a cumulative direct genetic gain by 1.2 SD_A_ or 28 %, averaging 7 % per generation. Correlated genetic changes in survival were small (−0.09 to 0.264 SD_A_).

**Table 4:** Heritability, maternal and common environmental effects for body weight and survival and phenotypic (r_p_) and genetic (r_g_) correlations between the two traits

Traits	Heritability	Full-sib effects	r_g_	r_p_
Weight	0.17 ± 0.05	0.16 ± 0.02	0.10 ± 0.08	−0.03 ± 0.01
Survival	0.05 ± 0.01^a^			
	0.17 ± 0.03^b^			

^a^ Estimated from animal mixed model, ^b^ estimated from threshold logistic model

**Table 5:** Genetic gain calculated as the difference in breeding values between the selection line and control in actual unit of measurements or expressed in genetic standard deviation (SD_A_) unit

Generation	Weight		Survival	
	Actual unit	Genetic SD	Actual unit	Genetic SD
G1	0.07	0.0008	0.0468	0.485
G2	46.61	0.495	0.0016	0.017
G3	84.31	0.895	0.0127	0.132
G4	18.23	0.194	0.0198	0.206

**Table 6:** Genetic gain calculated as the difference in breeding values of the selection line between successive generations in actual unit of measurements or expressed in genetic standard deviation unit

Generation	Weight		Survival	
	Actual unit	Genetic SD	Actual unit	Genetic SD
G1	0.07	0.0008	0.0027	0.027
G2 – G1	39.53	0.420	0.0180	0.186
G3 – G2	34.33	0.365	0.0254	0.264
G4 – G3	7.43	0.079	0.0006	0.006

## Discussion

### Selection response and future improvement

Genetic evaluation of 17,351 animals produced from 342 sires and 352 dams over four generations between 2004 and 2014 demonstrated that the selection program resulted in a remarkable improvement in growth performance in the common carp population. The average genetic gain was approximately 7 % per generation (2 years per generation). Our results are in good agreement with those reported recently for common carp by Ninh et al. [9] and Vandeputte et al. [8] as well as other aquatic animal species such as tilapia [12] and giant freshwater prawn [13]. Across farmed aquaculture species, direct response to selection for increased body weight or high growth range from 5–15 % per generation [5, 7]. Estimates of genetic gain depend on statistical methods used or whether a comparison is made with a control (fish having breeding values close to the population mean) or with wild stocks. In the present study, a separate control group of the same genetic origin as the selection line was maintained and contemporaneously produced in each generation. The genetic gain was estimated using the difference in: i) EBVs between the two lines (method 1); and ii) EBV between successive generations (method 2). The magnitude of genetic gain obtained from methods one and two are similar. Hung et al. [13] reported that the genetic gain slightly differed by the two methods. The EBV is a measure of the genetic superiority in a trait of interest of an animal as compared to its contemporaries and is calculated from the phenotypes of the individual and pedigree data. Thus, an analysis of the genetic trend calculated using EBV provided a more accurate indication of the amount of genetic progress attained in selected populations for traits studied as compared to using the phenotypic means [14]. Our results, when considered with those reported by Hung et al. [13] and Hamzah et al. [12], suggest that genetic response can be estimated with a minimum bias by having a contemporary control in parallel with the selection line in all generations. However, in commercial breeding programs, where resources are not available to maintain a separate control, assessment of genetic progress in selection populations could be estimated as the differences in EBV between consecutive generations.

For aquaculture enterprises, body weight and survival rate are the two most important economic traits. We investigated these changes associated with the selection program for common carp. Using two estimation methods and four generations, changes in survival rate during grow-out were negligible. This is consistent with the absence of genetic correlation between body weight and survival (Table 4). Correlated changes in survival to selection for increased body weight of common carp have not been reported previously but the non-significant changes in survival during grow-out in the present population are in agreement with reports in other freshwater fish, e.g. tilapia [15, 16]. There is a growing concern about negative changes in fitness related traits in artificial selection programs [5]. However, the non-significant correlated response in survival shown in our research indicates that selection for high growth did not have detrimental effect on fitness in the common carp population over a period of ten years.

Although survival was not improved from the selection program for high growth, this trait has a heritable additive genetic component (heritability = 0.05 to 0.17), indicating that direct selection to improve survival, albeit at a slow rate, could be possible in practical genetic improvement programs for common carp. A number of studies report that index selection, combining growth and survival, resulted in the improvement in both traits in Pacific white leg shrimp *Liptopenaus vannamei* [17], tilapia *Oreochromis aureus* [18] and abalone *Haliotis diversicolor* [19]. Expanding the breeding objectives for common carp by including new traits (especially survival rate or disease resistance) is currently under investigation in our breeding program.

The heritability of body weight was shown to be moderate and significant in our study and indicates that this population of common carp will continue to show a positive response to future selection. Our estimate of heritability for body weight is consistent with studies reported recently in common carp [20–23] and other fishes [12] as well as in crustacean species such as shrimp [24, 25] and mollusc, e.g. abalone [19]. Vandeputte [26] reported that the heritability range for body weight in common carp was 0.00 to 0.75. An evaluation of the literature, together with the heritability estimate obtained from our present study, suggest that selective breeding is an effective way of improving growth related traits.

### Common full-sib effects

It is not unsurprising that the common full-sib effect (c^2^) accounted for about 17 % of the total phenotypic variance for body weight. This is mainly a result of the separate early rearing of each family for 2–3 months before tagging. Under separate family rearing in a different selected population of common carp, Ninh et al. [20] reported that the c^2^ effect ranged from 0.11 to 0.30. A large proportion of common full-sib variance was also reported for body traits in other finfishes such as tilapia [27], Atlantic salmon [28], rainbow trout [29], and giant freshwater prawn [25, 30]. Early communal rearing of all families soon after birth can reduce the c^2^ effect as reported across aquaculture species, including common carp [20] and marine yellowtail kingfish [31].

### On farm testing of the improved strain

On farm testings of the improved common carp strain were carried out in several different geography and climate locations covering east China (Jiangsu and Shandong Provinces), southwest China (Sichuan Province and Guizhou Province), northwest China (Gansu Province and Ningxia Province) and northeast China (Liaoning Province). Compared with local common carp varieties, our improved strain had greater growth performance (20.1–39.2 %), higher survival (1.0–8.9 %) and lower food conversion rate (8.5–22.8 %) across all the regions (Additional file 3). These results are in good agreement with those reported in other Asian countries, showing that improved carp strains are superior to local stocks across different farming systems in Bangladesh, Thailand and Vietnam [32]. In India, Mahapatra et al. [33] reported that the improved rohu carp had 96 % greater body weight than the stock of farmers. The superiority of our improved common carp strain, under both selection and production environments, demonstrate that the genetic progress achieved in the nucleus is also expressed effectively under practical conditions and that the use of the improved strain can help farmers/producers to accelerate commercial production.

### Existing challenges

Further to the successful outcome from the selective breeding program for common carp in the present population, there are challenges for the long-term success in genetic improvement for this species. For instance, induced breeding at the first sexual maturation of the females did not result in 100 % success rate of spawning. A second injection was needed for females that failed to spawn the first time, causing a delay of one to two weeks compared with the normal reproduction for culture production. In all generations, offspring from all families were reared separately in hapas (net cages). The use of outdoor net cages installed in a pond for family rearing has proven successful, but the growth and survival rates of the fish in the early stages of development, were lower than for those reared under normal pond conditions. The fine mesh (8–16 mesh per cm) of the cage restricted water exchange and supply of natural food into the cage, which had an adverse impact on the growth and survival of the fish. It was also difficult to maintain a uniform culture environment for different families contained in separate cages, and to ensure a normal rate of growth and development when there are limitations with the physical rearing facilities. However, using individual earthen ponds for family rearing was not feasible due to the large number of families and cages were used for the family rearing in all generations. One option to overcome these limitations is to apply DNA markers for parentage assignment to enable the early communal rearing of all families after birth, as demonstrated in common carp [9]. It is also necessary to record growth and survival traits during the early phase of rearing in order to allow a formal genetic evaluation for these characters in efforts to broaden the breeding objectives in the present common carp population.

## Conclusion

Four generations of selection for increased harvest body weight achieved an average direct genetic gain of approximately 7 % per generation. The selection program did not have a negative impact on survival rate of the animal over the ten year research period. The moderate heritability for body weight indicates that the population will respond to future selection. The significant additive genetic component for survival provides the possibility of further improving this characteristic in the future selective breeding program for *C. carpio*.

## Additional files

Additional file 1: Table S1. Schedule of reproduction and management. (DOC 30 kb) Additional file 2: Table S2. Significance of fixed effects and covariate for body weight and survival. (DOC 30 kb) Additional file 3: Table S3. Growth performance from on-farm testing experiments of the improved common carp compared with local variety. (DOC 42 kb)

## References

[1] FAO (2012). The state of world fisheries and aquaculture.

[2] TFBMA. China yearbook of fishery statistics. The Fishery Bureau of Ministry of Agriculture. Beijing, Chinese Agriculture Press. 2012.

[3] Balon EK. Origin and domestication of the wild carp, *Cyprinus carpio*: from Roman gourmets to the swimming flowers. Aquaculture. 1995;129(1):3–48.

[4] Dong Z, Yuan X. The utilizations of heterosis in common carp in China. Aquaculture Asia. 2002;7(2):14-15.

[5] Nguyen HN. Genetic improvement for important farmed aquaculture species with a reference to carp, tilapia and prawns in Asia: achievements, lessons and challenges. Fish and Fisheries 2015:10.1111/faf.12122.

[6] Gianola D, Rosa GJ (2015). One hundred years of statistical developments in animal breeding. Annu Rev Anim Biosci.

[7] Gjedrem T (2012). Genetic improvement for the development of efficient global aquaculture: a personal opinion review. Aquaculture.

[8] Vandeputte M, Kocour M, Mauger S, Rodina M, Launay A, Gela D, et al. Genetic variation for growth at one and two summers of age in the common carp (*Cyprinus carpio *L.): Heritability estimates and response to selection. Aquaculture. 2008;277:7–13.

[9] Ninh NH, Ponzoni RW, Nguyen NH, Woolliams JA, Taggart JB, McAndrew BJ (2013). A comparison of communal and separate rearing of families in selective breeding of common carp (*Cyprinus carpio*): Responses to selection. Aquaculture.

[10] Nguyen N, Whatmore P, Miller A, Knibb W. Quantitative genetic properties of four measures of deformity in yellowtail kingfish *Seriola lalandi *Valenciennes, 1833. J Fish Dis. 2015. doi:10.1111/jfd.12348. 10.1111/jfd.12348 25683477

[11] Gilmour AR, Gogel B, Cullis B, Thompson R, Butler D (2009). ASReml user guide release 3.0.

[12] Hamzah A, Ponzoni RW, Nguyen NH, Khaw HL, Yee HY, Nor SAM (2014). Genetic evaluation of the Genetically Improved Farmed Tilapia (GIFT) strain over ten generations of selection in Malaysia. J Trop Agr Sci.

[13] Hung D, Vu NT, Nguyen NH, Ponzoni RW, Hurwood DA, Mather PB (2013). Genetic response to combined family selection for improved mean harvest weight in giant freshwater prawn (*Macrobrachium rosenbergii*) in Vietnam. Aquaculture.

[14] Nicholas FW. Introduction to veterinary genetics: John Wiley & Sons; 2009.

[15] Hamzah A, Mekkawy W, Khaw HL, Nguyen NH, Yee HY, Bakar KRA, et al. Genetic parameters for survival during the grow out period in the GIFT strain and correlated response to selection for body weight. West Sussex, United Kingdom: Aquaculture Research 2015; accepted.

[16] Ninh NH, Thoa NP, Knibb W, Nguyen NH (2014). Selection for enhanced growth performance of Nile tilapia (*Oreochromis niloticus*) in brackish water (15–20 ppt) in Vietnam. Aquaculture.

[17] Gitterle T, Johansen H, Erazo C, Lozano C, Cock J, Salazar M, et al. Response to multi-trait selection for harvest weight, overall survival, and resistance to white spot syndrome virus (WSSV) in *Penaeus (Litopenaeus) vannamei*. Aquaculture. 2007;272:S262.

[18] Thodesen J, Rye M, Wang Y-X, Li S-J, Bentsen HB, Gjedrem T. Genetic improvement of tilapias in China: Genetic parameters and selection responses in growth, pond survival and cold-water tolerance of blue tilapia (*Oreochromis aureus*) after four generations of multi-trait selection. Aquaculture. 2013;396–399:32–42.

[19] Liu J, Lai Z, Fu X, Wu Y, Bao X, Hu Z, et al. Genetic parameters and selection responses for growth and survival of the small abalone *Haliotis diversicolor *after four generations of successive selection. Aquaculture. 2015;436:58–64.

[20] Ninh NH, Ponzoni RW, Nguyen NH, Woolliams JA, Taggart JB, McAndrew BJ (2011). A comparison of communal and separate rearing of families in selective breeding of common carp (*Cyprinus carpio*): estimation of genetic parameters. Aquaculture.

[21] Vandeputte M, Kocour M, Mauger S, Dupont-Nivet M, De Guerry D, Rodina M, et al. Heritability estimates for growth-related traits using microsatellite parentage assignment in juvenile common carp (*Cyprinus carpio *L.). Aquaculture. 2004;235(1):223–36.

[22] Kocour M, Mauger S, Rodina M, Gela D, Linhart O, Vandeputte M. Heritability estimates for processing and quality traits in common carp (*Cyprinus carpio *L.) using a molecular pedigree. Aquaculture. 2007;270(1):43–50.

[23] Nielsen HM, Ødegård J, Olesen I, Gjerde B, Ardo L, Jeney G (2010). Genetic analysis of common carp (*Cyprinus carpio*) strains: I: Genetic parameters and heterosis for growth traits and survival. Aquaculture.

[24] Nguyen NH, Quinn J, Powell D, Elizur A, Thoa NP, Nocillado J, et al. Heritability for body colour and its genetic association with morphometric traits in Banana shrimp (*Fenneropenaeus merguiensis*). BMC Genet. 2014;15(1):132. 10.1186/s12863-014-0132-5 PMC4261751 25476506

[25] Hung D, Nguyen NH, Hurwood DA, Mather PB (2014). Quantitative genetic parameters for body traits at different ages in a cultured stock of giant freshwater prawn (*Macrobrachium rosenbergii*) selected for fast growth. Mar Freshw Res.

[26] Vandeputte M. Selective breeding of quantitative traits in the common carp (Cyprinus carpio): a review. Aquat Living Resour. 2003;16(05):399–407.

[27] Nguyen NH, Ponzoni RW, Abu-Bakar KR, Hamzah A, Khaw HL, Yee HY. Correlated response in fillet weight and yield to selection for increased harvest weight in genetically improved farmed tilapia (GIFT strain), *Oreochromis niloticus*. Aquaculture. 2010;305(1–4):1–5.

[28] Yáñez JM, Lhorente JP, Bassini LN, Oyarzún M, Neira R, Newman S. Genetic co-variation between resistance against both *Caligus rogercresseyi *and *Piscirickettsia salmonis*, and body weight in Atlantic salmon (*Salmo salar*). Aquaculture. 2014;433:295–8.

[29] Haffray P, Bugeon J, Rivard Q, Quittet B, Puyo S, Allamelou JM, et al. Reprint of: Genetic parameters of in-vivo prediction of carcass, head and fillet yields by internal ultrasound and 2D external imagery in large rainbow trout (*Oncorhynchus mykiss*). Aquaculture. 2014;420:S134–42.

[30] Hung D, Nguyen HN (2014). Genetic inheritance of female and male morphotypes in giant freshwater prawn *Macrobrachium rosenbergii*. PLoS One.

[31] Whatmore P, Nguyen NH, Miller A, Lamont R, Powell D, D’Antignana T (2013). Genetic parameters for economically important traits in yellowtail kingfish *Seriola lalandi*. Aquaculture.

[32] Nguyen NH, Ponzoni RW. Genetic improvement of carp reduces poverty and hunger in Asia. Global Aquaculture Advocate 2008:76–78.

[33] Mahapatra KD, Saha J, Sarangi N, Jana R, Gjerde B, Nguyen N, et al. Genetic improvement and dissemination of Rohu (*Labeo rohita*, Ham.) in India. In: Genetic improvement: making it happen. Proceedings of the Seventeenth Conference of the Association for the Advancement of Animal Breeding and Genetics: 23rd-26th September. Armidale, N.S.W. Australia Armidale, New South Wales, Australia: Association for the Advancement of Animal Breeding and Genetics; 2007;17:37–40.

