# Supplementary material for: Genetic evaluation of a selective breeding program for common carp Cyprinus carpio conducted from 2004 to 2014
Source: BMC Genet. 2015 Jul 29;16:94. doi: 10.1186/s12863-015-0256-2 (PMC4518635; doi:10.1186/s12863-015-0256-2)
Supplement: Additional file 1: Table S1. — Schedule of reproduction and management. (DOC 30 kb) [file 12863_2015_256_MOESM1_ESM.doc]

**Additional file 1: Table S1.** Schedule of reproduction and management

| **Activities** | **G1** | **G2** | **G3** | **G4** |
| --- | --- | --- | --- | --- |
| Mating | 16 April 2004 | April 2006 | April 2008 | April 2012 |
| Nursing to 3 mm | May – June 2004 | May – June 2006 | May – June 2008 | May – June 2012 |
| Rearing to 20 mm | July – 15 August 2004 | July – August 2006 | July – August 2008 | July – August 2012 |
| Tagging | September 2004 | September 2006 | September 2008 | September 2012 |
| Growing out | October 2004 – Nov 2005 | October 2006 – Nov 2007 | October 2008 – Nov 2009 | August 2012 – Dec 2013 |
| Harvest | November 2005 | November 2007 | November 2009 | December 2013 |
