# Supplementary material for: Genetic evaluation of a selective breeding program for common carp Cyprinus carpio conducted from 2004 to 2014
Source: BMC Genet. 2015 Jul 29;16:94. doi: 10.1186/s12863-015-0256-2 (PMC4518635; doi:10.1186/s12863-015-0256-2)
Supplement: Additional file 2: Table S2. — Significance of fixed effects and covariate for body weight and survival. (DOC 30 kb) [file 12863_2015_256_MOESM2_ESM.doc]

Additional file 2: Table S2. Significance of fixed effects and covariate for body weight and survival

| Effect | DF | F-value | |
| --- | --- | --- | --- |
|  |  | Weight | Survival |
| Generation | 3 | 1420.8*** | 0.02 ^ns^ |
| Line | 1 | 93.5*** | 0 ^ns^ |
| Sex | 1 | 214.9*** | n.e. |
| Gen×Line | 3 | 41.6*** | 0 ^ns^ |
| Generation×Sex | 3 | 11.8*** | n.e. |
| Line×Sex | 1 | 0.7 ^ns^ | n.e. |
| Stock weight(Generation, Line) | 4 | 874.7*** | 0.08 ^ns^ |

n.e. = not included in the model as sex of dead fish was not known
