# Supplementary material for: Genetic evaluation of a selective breeding program for common carp Cyprinus carpio conducted from 2004 to 2014
Source: BMC Genet. 2015 Jul 29;16:94. doi: 10.1186/s12863-015-0256-2 (PMC4518635; doi:10.1186/s12863-015-0256-2)
Supplement: Additional file 3: Table S3. — Growth performance from on-farm testing experiments of the improved common carp compared with local variety. (DOC 42 kb) [file 12863_2015_256_MOESM3_ESM.doc]

Additional file 3: Table S3. Growth performance from on-farm testing experiments of the improved common carp compared with local variety

| Traits | Location | Improved carp strain | | Local stock of farmers | | Superiority (%) |
| --- | --- | --- | --- | --- | --- | --- |
|  |  | n | LSM±SE | n | LSM±SE |  |
| Weight (g) | Pi County, Sichuan Province | 100 | 699.47±121.14 | 100 | 582.58±145.75 | 20.1 |
|  | Dongping County, Shandong Province | 100 | 400.80±95.86 | 100 | 308.44±79.08 | 29.9 |
|  | Tongshan County, Jiangsu Province | 100 | 665.70±141.90 | 100 | 478.26±101.86 | 39.2 |
|  | Dawa County, Liaoning Province | 300 | 593.57±132.71 | 300 | 480.13±105.62 | 23.6 |
|  | Suiyang County, Guizhou Province | 500 | 286.15±46.17 | 500 | 221.60±40.28 | 29.1 |
|  | Yongjing County, Gansu Province | 500 | 1061.1±217.38 | 500 | 789.5±186.74 | 34.4 |
|  | Pingluo County, Ningxia Province | 200 | 847.53±170.39 | 200 | 665.67±140.29 | 27.4 |
|  | Yinchuan City, Ningxia Province | 200 | 1060.50±221.42 | 200 | 812.36±181.35 | 30.6 |
| Survival (%) | Suiyang County, Guizhou Province | 1500 | 91.9 | 1500 | 87.7 | 4.2 |
|  | Yongjing County, Gansu Province | 3000 | 95.2 | 3000 | 94.2 | 1.0 |
|  | Yin Chuan, Ninxia Province | 2000 | 94.7 | 2000 | 85.8 | 8.9 |
| FCR (unit) | Suiyang County, Guizhou Province | 1500 | 1.36 | 1500 | 1.67 | 22.8 |
|  | Pingluo County, Ningxia Province | 3000 | 1.65 | 3000 | 1.79 | 8.5 |

Survival was calculated as percent difference in the number of fish between stocking and final harvest, and FCR = Food conversion ratio (total feed intake/weight gain)
